# Differentiating the lung lesions using Intravoxel incoherent motion diffusion-weighted imaging: a meta-analysis

**DOI:** 10.1186/s12885-020-07308-z

**Published:** 2020-08-24

**Authors:** Jianye Liang, Jing Li, Zhipeng Li, Tiebao Meng, Jieting Chen, Weimei Ma, Shen Chen, Xie Li, Yaopan Wu, Ni He

**Affiliations:** 1 Department of Medical Imaging, Sun Yat-sen University Cancer Center, State Key Laboratory of Oncology in South China, Collaborative Innovation Center for Cancer Medicine, No.651, Dongfeng Road East, Guangzhou, 510060 Guangdong China; 2 Department of Radiology, Maoming People’s Hospital, Maoming, 525400 Guangdong China

**Keywords:** IVIM-DWI, Post-test probability, Diagnostic performance, Lung neoplasm, Magnetic resonance imaging, Meta-analysis

## Abstract

**Background and objectives:**

The diagnostic performance of intravoxel incoherent motion diffusion-weighted imaging (IVIM-DWI) in the differential diagnosis of pulmonary tumors remained debatable among published studies. This study aimed to pool and summary the relevant results to provide more robust evidence in this issue using a meta-analysis method.

**Materials and methods:**

The researches regarding the differential diagnosis of lung lesions using IVIM-DWI were systemically searched in Pubmed, Embase, Web of science and Wangfang database without time limitation. Review Manager 5.3 was used to calculate the standardized mean difference (SMD) and 95% confidence intervals of apparent diffusion coefficient (ADC), tissue diffusivity (D), pseudo-diffusivity (D*), and perfusion fraction (f)**.** Stata 12.0 was used to pool the sensitivity, specificity, and area under the curve (AUC), as well as publication bias and heterogeneity. Fagan’s nomogram was used to predict the post-test probabilities.

**Results:**

Eleven studies with 481 malignant and 258 benign lung lesions were included. Most include studies showed a low to unclear risk of bias and low concerns regarding applicability. Lung cancer demonstrated a significant lower ADC (SMD = -1.17, *P* < 0.001), D (SMD = -1.02, P < 0.001) and f values (SMD = -0.43, *P* = 0.005) than benign lesions, except D* value (SMD = 0.01, *P* = 0.96). D value demonstrated the best diagnostic performance (sensitivity = 89%, specificity = 71%, AUC = 0.90) and highest post-test probability (57, 57, 43 and 43% for D, ADC, f and D* values) in the differential diagnosis of lung tumors, followed by ADC (sensitivity = 85%, specificity = 72%, AUC = 0.86), f (sensitivity = 71%, specificity = 61%, AUC = 0.71) and D* values (sensitivity = 70%, specificity = 60%, AUC = 0.66).

**Conclusion:**

IVIM-DWI parameters show potentially strong diagnostic capabilities in the differential diagnosis of lung tumors based on the tumor cellularity and perfusion characteristics, and D value demonstrated better diagnostic performance compared to mono-exponential ADC**.**

## Introduction

Lung cancer is the most commonly diagnosed cancer (11.6% of the total cases) and the leading cause of cancer death (18.4% of the total cancer deaths) in 2018 around the world [1]. The incidence and mortality of lung cancer still increased in recent 30 years. Accurate and early diagnosis is help to select optimal treatment strategy and improve the outcome of patients with lung cancer.

Computed tomography (CT) is the main imaging modality for lung lesions largely based on morphological and enhanced characteristics. However, the relatively low specificity and administration of contrast agent limit its wide use in clinical practice. Magnetic resonance imaging (MRI) was rarely used in detecting lung lesions previously due to the obvious cardiac and respiratory motion, low signal-to-noise ratio from the inherently low lung-proton density, and magnetic susceptibility artifact of air-filled pulmonary tissue subjected to high field strength [2]. With the development of MRI hardwares and various rapid imaging technologies such as improved gradient performance, parallel imaging techniques and free-breathing acquisition, MRI has been increasingly used for identification of benign and malignant lung tumors and efficacy evaluation. Diffusion-weighted imaging (DWI) is a radiation-free and contrast-free functional imaging sequence, which allows measurement of water molecular movement using apparent diffusion coefficient (ADC) and demonstrates potential to differentiate malignant from benign lung lesions. A previous meta-analysis even reported a higher diagnostic performance with a pooled sensitivity, specificity and areas under the curve (AUC) of 83, 91% and 0.93 in DWI, compared to PET/CT whose sensitivity, specificity and AUC were 78, 81% and 0.86, respectively. The mono-exponential model is expressed as SI / SI_0_ = exp(−b**·**ADC), where SI_0_ refers to the mean signal intensity (SI) of the region of interest for b = 0 s/mm^2^ while SI refers to the signal intensity for higher b values. However, the mono-exponential model cannot separate the pseudo-diffusion from pure molecular diffusion, and ADC calculated from the mono-exponential model mixes the two effects. Therefore, the conventional mono-exponential model cannot accurately reflect the true diffusivity owing to the influence of microcirculation perfusion [3].

Intravoxel incoherent motion (IVIM) is an advanced imaging technique, which was first proposed by Le Bihan et al. [4]. It can separate the incoherent motion of water molecules within the capillaries from molecular diffusion in the extravascular space [5]. The true diffusion coefficient (D value), pseudo-diffusion coefficient (D* value) and perfusion fraction (f value) were generated using a biexponential model with multiple b-values expressed as SI / SI_0_ = (1- f) **·** exp(−bD) + f **·** exp(−bD*)**.** The IVIM model can separate the pseudo-diffusion from pure molecular diffusion and independently reflect the microcirculation perfusion (D*) and tumor cellularity (D) based on that equation [6]. This model provides more detailed and accurate information, and can make a better interpretation for the microenvironment changes and characterization of tumor grades. As such, these parameters are important to be analyzed. Several studies had applied IVIM-DWI to discriminate lung cancer from benign lesions and demonstrated better or comparable diagnostic performance compared with traditional ADC value [7–9]. However, the diagnostic performances of IVIM-DWI derived parameters in the differentiation of lung tumors were not consistent and the application still remained debatable in the lung. For example, several studies indicated that lung cancer had a higher D* value than benign lesion [10–12] while some studies reported adverse [7, 8, 13] or insignificant results [9, 14, 15]. Theoretically, the true diffusitivity should have better diagnostic performance than ADC in distinguishing lung lesions, but some studies indicated a much lower area under the curve (AUC) or accuracy in D value compared to ADC [7, 14]. Cancerous tissue generally has active angiogenesis and rich blood supply compared to benign lesions, but most studies indicated a lower f value in lung cancer, the results of which should be further confirmed. The sample sizes in most studies were still not enough to draw a robust conclusion for its performance; the application of IVIM-DWI in the lung has not yet formed a clinical guideline or become a routine sequence in the MRI protocol. Therefore, we attempted to pool all the published results about the diagnostic performance of IVIM-DWI in the differentiation of malignant and benign lung lesions using a meta-analysis method. Besides, the diagnostic performance of IVIM-DWI was compared to conventional DWI-derived ADC value to determine the suitability for clinical application. The controversial issues between different researches will also be addressed with more reliable evidence. Furthermore, this study provides additional information about technical feasibility on lung MRI, and the functional changes of lung lesions with IVIM-DWI. This study may further attract the researchers to perform the lung studies using noninvasive MR imaging by solving the technical issues on Lung MRI.

## Materials and methods

### Data sources

The studies regarding the differential diagnosis of lung tumors using IVIM-DWI parameters were systemically retrieved by two senior librarians in PubMed, Embase, Web of science and Wangfang database without time limitation. A searching formula was formed with different combinations of the medical subject headings or key words from IVIM, intravoxel incoherent motion, multiple b-value DWI, biexponential, and lung or pulmonary lesion / cancer / carcinoma / neoplasm. The primary searches were limited in the titles and abstracts. We also performed a manual retrieval of the reference lists from included studies.

### Studies selection

Studies met the following criteria were included: (a) the research purpose was to differentiate lung cancer from benign lesions using IVIM-DWI parameters; (b) the mean and standard deviation (SD) of each parameter was provided; (c) their diagnostic performance about sensitivity and specificity, or true-positive (TP), false-negative (FN), false-positive (FP) and true-negative (TN) were reported; (d) the lung cancer should be confirmed by pathology after initial MRI examination. Exclusion criteria mainly included: (a) duplication from the same authors or institutions; (b) meta-analysis, conference abstract, review or any unpublished results; and (c) animal experiments or non-lung researches.

### Data extraction

A spreadsheet was used to extract the mean values and SD as well as the diagnostic performance of ADC, D, D* and f values with threshold value, AUC, sensitivity and specificity in respective study by one author, and reviewed by another one. Other information included the first author, publication years, field strength and vendors, b values, patient ages, tumor sizes, and numbers of malignant and benign lesions. TP, FN, FP and TN can be calculated when only the amount of malignant and benign lesions as well as sensitivity and specificity or receiver operating curve was provided.

### Quality assessment

The quality of studies and likelihood of bias were evaluated using Review Manager 5.3 software (Cochrane Collaboration, Oxford, UK), referring to the Quality Assessment of Diagnostic Accuracy Studies- 2 [16]. We assessed the risk of bias and applicability in four domains, including patient selection, index tests, reference standard, flow and timing [17].

### Publication bias and heterogeneity evaluation

As two parts of data were pooled in our study including quantitative values and diagnostic performance of each parameter, funnel plots and Begg’s test were used to visually and quantitatively assess the publication bias for the continuous variables and Deek’s plot assessed the publication bias of sensitivity and specificity using Stata version 12.0 (StataCorp LP, College Station, TX). An asymmetric or skewed funnel plot, *P* < 0.05 of Begg’s test or Deek’s test indicated the potential of publication bias [18]. Inconsistency index (I^2^) and Cochran’s Q tests were used to explore the heterogeneity of included studies, with I^2^ > 50% or *P* < 0.05 for Cochran Q test suggested statistically significant heterogeneity, and a random-effect model was applied in subsequent pooling, or a fixed-effect model when I^2^ < 50% [19].

### Evidence synthesis

We constructed the forest plots for continuous variables and calculated the standardized mean difference (SMD) between lung cancer and benign lesions using Review Manager software. We used the bivariate regression model to pool the diagnostic performance with sensitivity, specificity, positive likelihood ratio (PLR), negative likelihood ratio (NLR), diagnostic odds ratio (DOR) and AUC using Stata version 12.0. The summary receiver operating characteristic curves and Fagan’s nomograms were also plotted to determine the diagnostic values and predict the post-test probabilities of ADC, D, D* and f values in the differential diagnosis of lung tumors.

## Results

### Literature search and selection

**Fig. 1:**
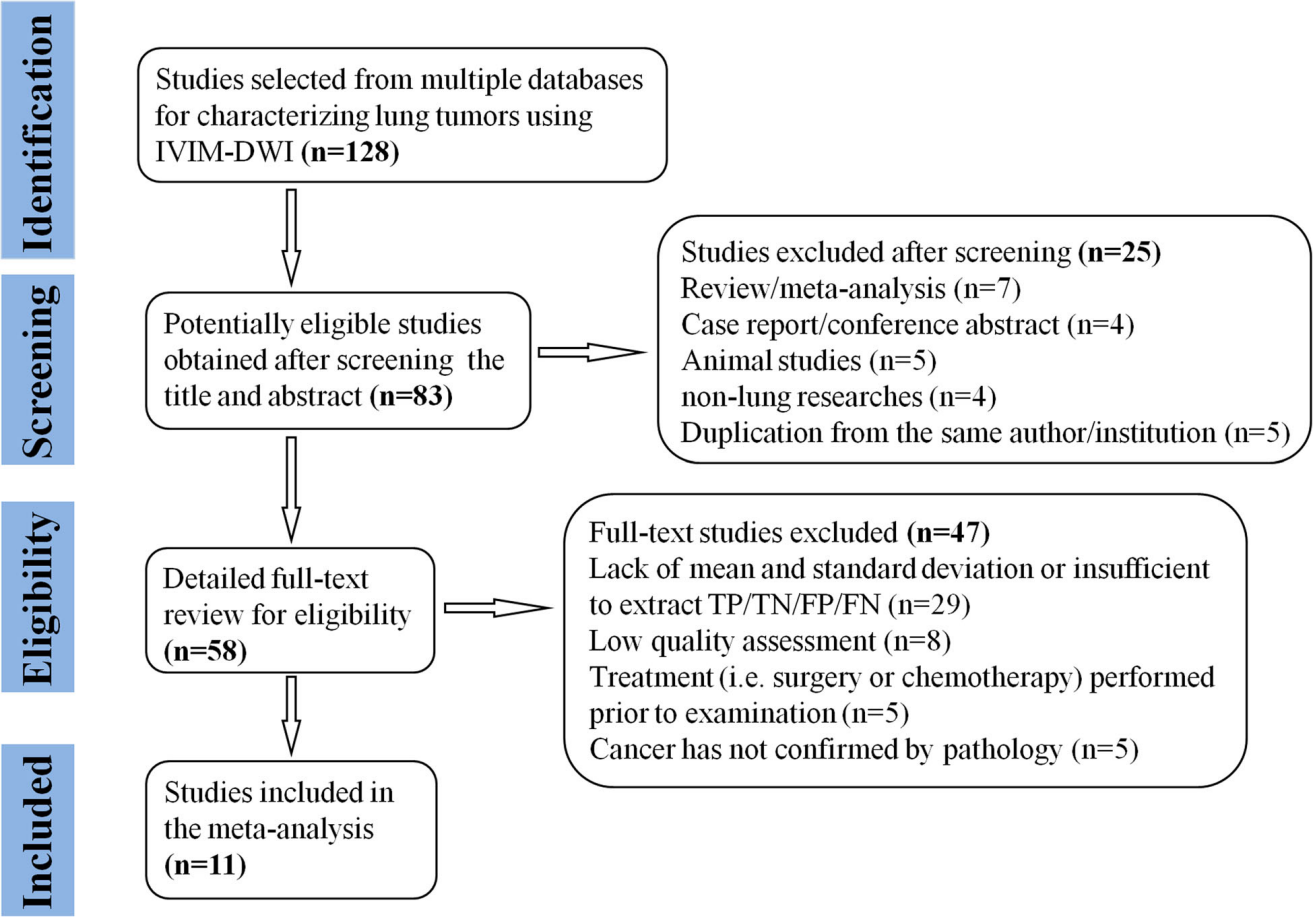
Flowchart detailing the study selection process. Eleven studies that met the inclusion criteria were included. FN, false negative; FP, false positive; TN, true negative; TP, true positive

By searching the key words in the titles and abstracts, a total of 128 potential studies were obtained from multiple databases. A total of 11 studies regarding meta-analysis, conference abstract, case report and review were excluded after screening the titles and abstracts. Animal studies, non-lung researches and duplication from the same authors or institutions led to further exclude 14 studies. We scrutinized the full-texts of the remaining 58 studies in detail and excluded an additional 47 studies for the following reasons: (a) lack of sufficient data to be pooled; (b) low quality assessment; (c) IVIM-DWI was interfered by treatment and (d) cancer was not confirmed by pathology. Eventually, 11 eligible studies with 481 malignant and 258 benign lung lesions were included for analysis. The flowchart detailing the process of study selection was provided in Fig. 1. Basic information and diagnostic performance for each included study was detailed in Table 1 and Table 2. In other to include every potential article, we did not set a criterion on the field strength (1.5 T or 3.0 T). From Table 1, there are three studies using 1.5 T and eight studies using 3.0 T for imaging. Although field strength of 3.0 T is better for image quality, the results from 1.5 T scanner are also acceptable. Therefore, studies with either of field strengths are included for analysis.

**Table 1:** Basic information for each included study

Author	Year	Machine type	b values (s/mm^2^)	Age (years)	Tumor size (cm)	Malignant	Benign
Deng et al. [7]	2015	3 T Philips	0,25,50,75,100,200,400,600,800,1000	58.80 ± 10.93	3.21 ± 1.62	30	8
Huang et al. [13]	2016	3 T GE	0,10,25,50,100,200,400,600,800,1000	57.4 ± 13.2	NA	30	15
Jiang et al. [8]	2020	3 T Siemens	0,50,100,150,200,250,300,500,800,1000	60.2 (21–80)	3.72 ± 1.71	88	33
Jiao et al. [10]	2019	3 T GE	0,20,50,100,200,400,600,800,1000	38–79	NA	59	37
Wan et al. [9]	2018	3 T Philips	0,5,10,15,20,25,50,80,150,300,500,600,800,1000	58.25 (23–77)	4.2 (1.0–14.8)	69	20
Wang LL et al. [14]	2014	1.5 T Siemens	0,5,10,15,20,25,50,80,150,300,500,600,800	57.17 ± 8.82	2.89 ± 1.19	31	31
Wang Y et al. [11]	2019	3 T Philips	0,5,10,15,20,25,50,80,150,300,500,800,1000	33–79	NA	30	20
Yuan et al. [15]	2015	3 T Siemens	0,50,100,150,200,400,600,800	NA	2.9 (1.8–9.0)	52	48
Zhou et al. [12]	2018	1.5 T GE	0,20,50,100,150,200,400,600,1000	52.8 ± 10.5		42	22
Wang XH et al. [20]	2014	3 T GE	0,50,100,150,200,400,600,1000,1500	57.7 ± 12.7	5.2 ± 2.7	23	15
Koyama et al. [2]	2015	1.5 T Philips	0,50,100,150,300,500,1000	68.3 ± 10.2	0.4–7.33	27	9

NA Not available

**Table 2:** The diagnostic performance for each included study

Indicator	Author	Year	Threshold	AUC	Sensitivity	Specificity	TP	FP	FN	TN
ADC	Deng et al. [7]	2015	1.0224	0.833	0.733	0.875	22	1	8	7
	Huang et al. [13]	2016	1.547	0.805	0.889	0.667	27	5	3	10
	Jiang et al. [8]	2020	1.46	0.805	0.9245	0.6316	81	12	7	21
	Wan et al. [9]	2018	1.734	0.773	0.793	0.749	55	5	14	15
	Wang Y et al. [11]	2019	1.265	0.847	0.847	0.715	25	6	5	14
	Yuan et al. [15]	2015	1.31	NA	0.812	0.812	42	9	10	39
	Zhou et al. [12]	2018	1.57	0.708	0.905	0.591	38	9	4	13
D	Huang et al. [13]	2016	1.04	0.93	0.944	0.75	28	4	2	11
	Jiang et al. [8]	2020	1.23	0.882	0.9057	0.8947	80	3	8	30
	Jiao et al. [10]	2019	0.958	0.812	0.763	0.78	45	8	14	29
	Wan et al. [9]	2018	1.138	0.834	0.8551	0.75	59	5	10	15
	Wang LL et al. [14]	2014	0.98	0.763	0.871	0.665	27	10	4	21
	Wang Y et al. [11]	2019	1.185	0.888	0.888	0.752	27	5	3	15
	Yuan et al. [15]	2015	1.44	NA	0.913	0.385	47	30	5	18
	Zhou et al. [12]	2018	1.25	0.729	0.952	0.545	40	10	2	12
	Wang XH et al. [20]	2014	0.9	0.839	0.957	0.8	22	3	1	12
D*	Deng et al. [7]	2015	NA	0.679	0.622	0.8	19	2	11	6
	Huang et al. [13]	2016	17.935	0.605	0.765	0.462	23	8	7	7
	Jiang et al. [8]	2020	15.9	0.696	0.7925	0.6316	70	12	18	21
	Wan et al. [9]	2018	NA	NA	0.693	0.45	48	11	21	9
	Yuan et al. [15]	2015	12.71	NA	0.478	0.692	25	15	27	33
	Zhou et al. [12]	2018	8.82	0.68	0.714	0.591	30	9	12	13
	Wang XH et al. [20]	2014	3.7	0.683	0.826	0.6	19	6	4	9
f	Deng et al. [7]	2015	37.43%	0.829	0.8	0.75	24	2	6	6
	Huang et al. [13]	2016	28.35%	0.615	0.75	0.429	23	9	7	6
	Wan et al. [9]	2018	NA	NA	0.719	0.5	50	10	19	10
	Wang LL et al. [14]	2014	24.93%	0.762	0.806	0.548	25	14	6	17
	Yuan et al. [15]	2015	18.36%	NA	0.609	0.692	32	15	20	33
	Wang XH et al. [20]	2014	39.30%	0.639	0.521	0.8	12	3	11	12

*NA* Not available, *ADC* Apparent diffusion coefficient, ***D*** Tissue diffusivity, D* pseudo-diffusivity, *f* Perfusion fraction, *AUC* Area under the curve, *FN* False negative, *FP* False positive, *TN* True negative, *TP* True positive. Threshold values of ADC, D and D* are factors of 10^− **3**^ mm^**2**^/s

### Quality assessment

**Fig. 2:**
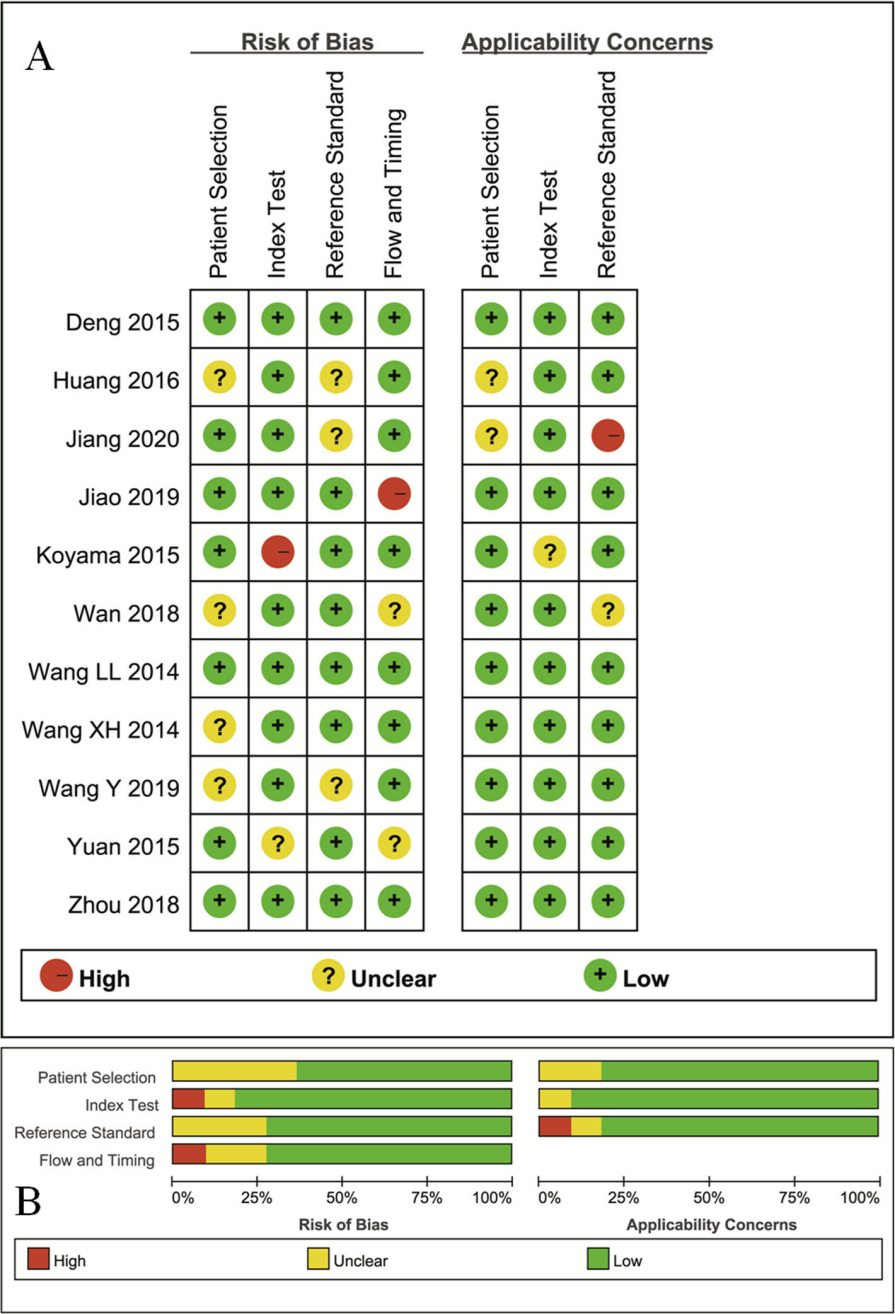
The distribution of risk of bias and applicability concerns for each included study using QUADAS-2 (**a**) and a summary methodological quality (**b**)

The distribution of Quality Assessment of Diagnostic Accuracy Studies–2 scores for risk of bias and applicability concerns were shown in Fig. 2. The overall quality of included studies was acceptable. Regarding patient selection, four studies were marked unclear risk of bias due to ambiguity for consecutive enrollment and prospective design or not. The applicability concerns remained unclear concern as the tumor types were inconsistent between malignant and benign tumors from two studies. Two studies were marked unclear and high risk of bias with unclear concern of applicability for index test as the threshold values for D* and f values were not provided. Three studies showed unclear risks of bias for reference standard because some of the benign lesions were diagnosed through a long time follow-up. Three studies were marked unclear and high risk of bias in patient flow and timing domain because the time interval between MR examination and pathological confirmation was not reported.

### Quantitative analysis

#### ADC used for diagnosis of lung tumor.

**Fig. 3:**
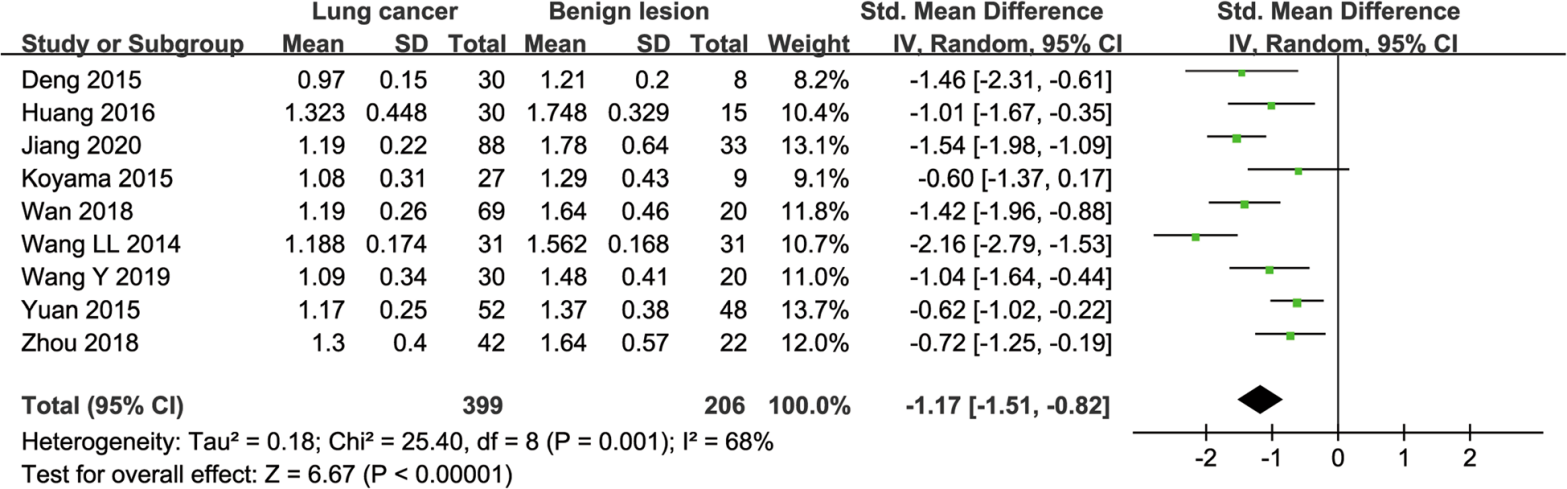
Forest plot of the mean value of apparent diffusion coefficient (**ADC**) between lung cancer and benign lesions. The standardized mean differences indicated that lung cancers had a significantly lower ADC than benign lesions

**Fig. 4:**
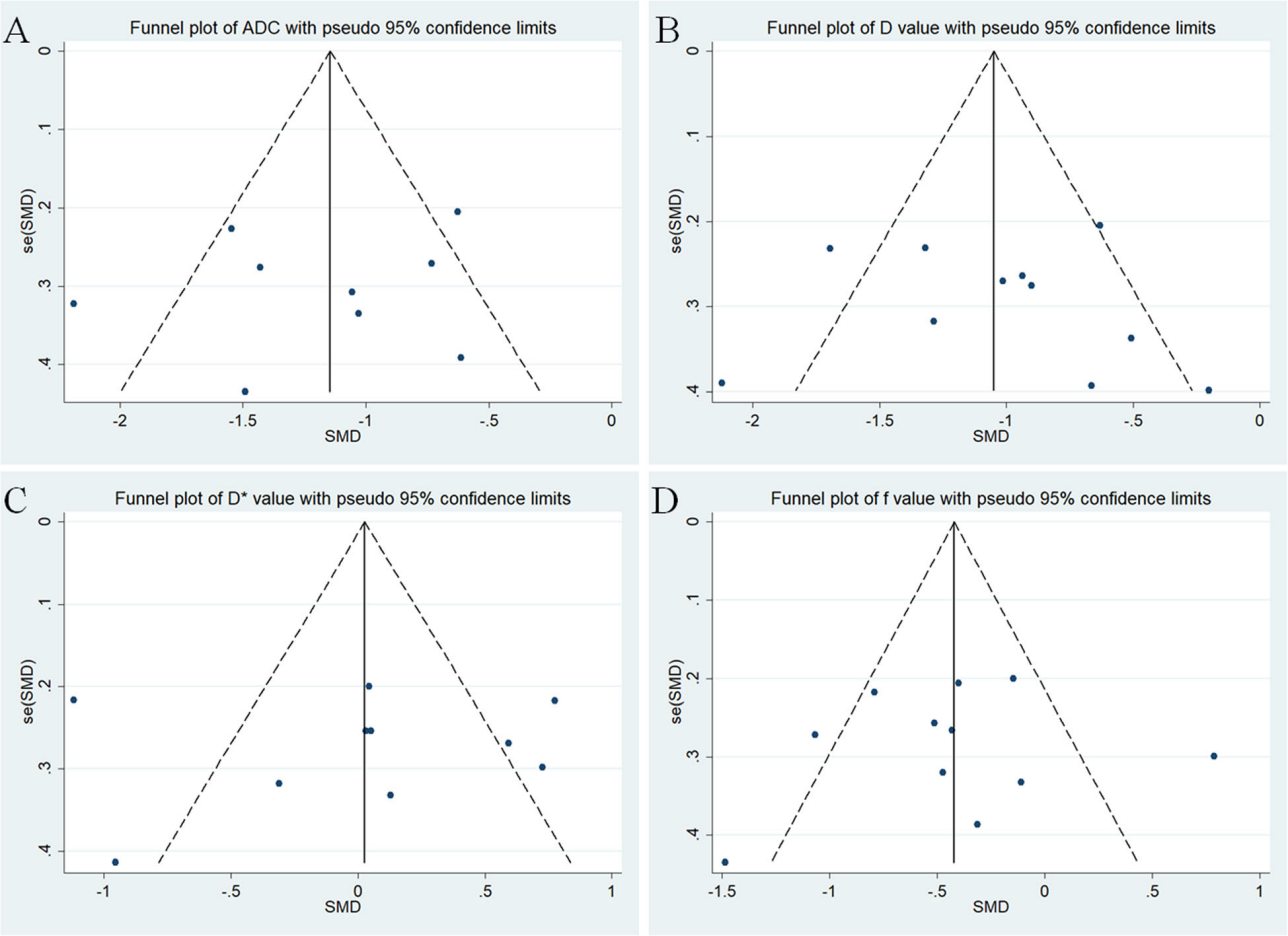
Funnel plot of **a** apparent diffusion coefficient (ADC), **b** tissue diffusivity (D), **c** pseudo-diffusivity (D*), and **d** perfusion fraction (f). The basically symmetric funnel plots indicated no publication bias in these parameters

Nine studies regarding ADC used in differentiating lung tumors were included for analysis. The χ^2^ = 25.40 and *P* = 0.001 of heterogeneity test with I^2^ = 68% suggested moderate heterogeneity among included studies. The forest plot in Fig. 3 showed the distribution of ADC between lung cancer and benign lesions. A random-effects model generated a SMD of − 1.17 (− 1.51, − 0.82) (*P* < 0.001) between lung cancer and benign lesions for ADC. A basically symmetric funnel plot in Fig. 4 and *P* = 0.754 of Begg’s Test suggested no publication bias in ADC.

#### D value used for diagnosis of lung tumor

**Fig. 5:**
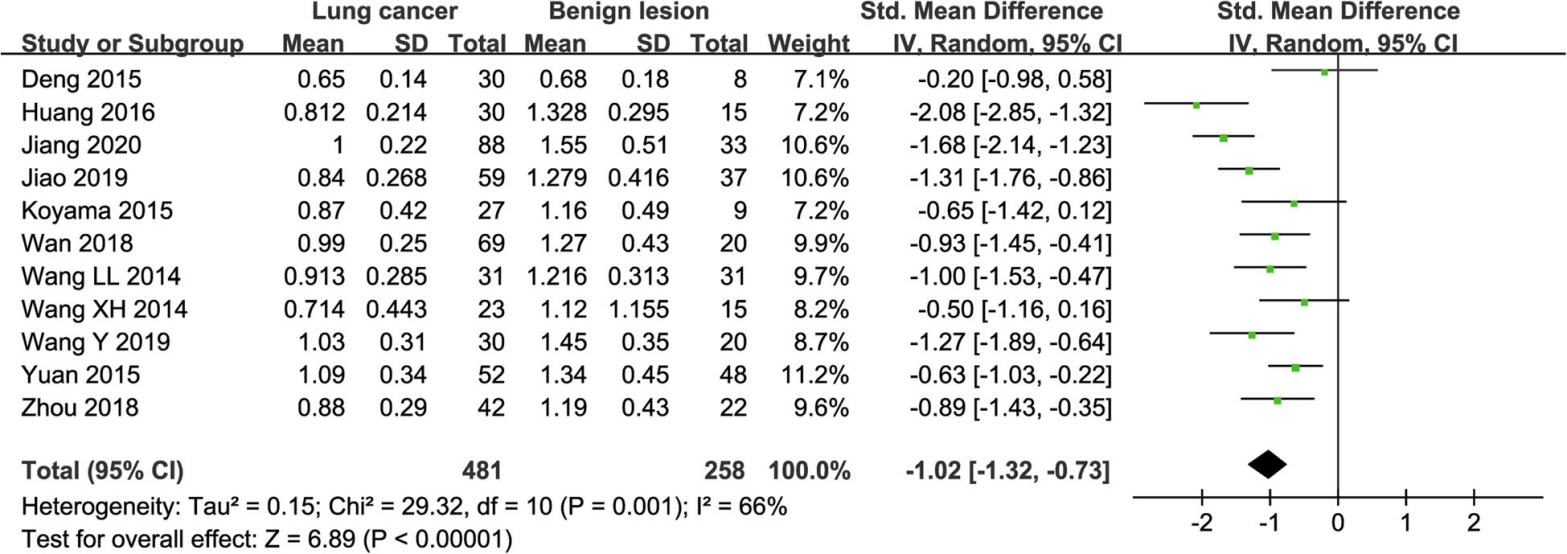
Forest plot of the mean value of tissue diffusivity (D) between lung cancer and benign lesions. The standardized mean differences indicated that lung cancer had a significantly lower D value than benign lesions

Eleven studies regarding D value used in differentiating lung tumors were included for analysis. The χ^2^ = 29.32 and *P* = 0.001 of heterogeneity test with I^2^ = 66% suggested moderate heterogeneity among included studies. The forest plot in Fig. 5 showed the distribution of D value between lung cancer and benign lesions. A random-effects model generated a SMD of − 1.02 (− 1.32, − 0.73) (*P* < 0.001) between lung cancer and benign lesions for D value. A basically symmetric funnel plot in Fig. 4 and *P* = 0.436 of Begg’s Test suggested no publication bias in D value.

#### D* value used for diagnosis of lung tumor

**Fig. 6:**
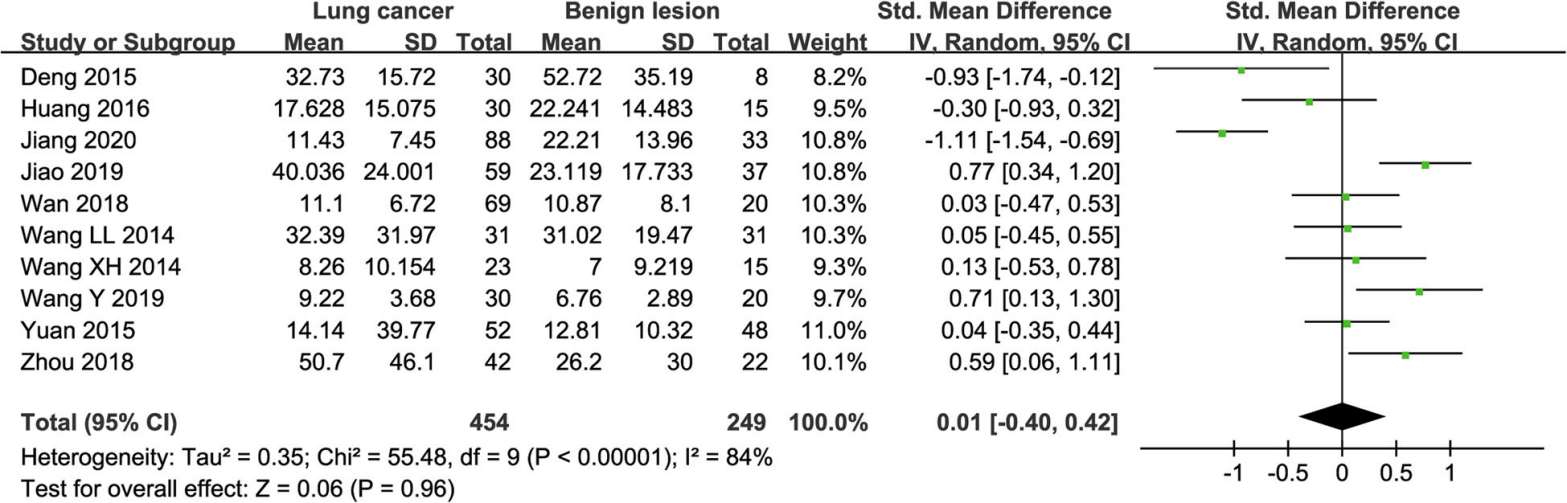
Forest plot of the mean value of pseudo-diffusivity (D*) between lung cancer and benign lesions. The standardized mean differences indicated that the difference of D* value between lung cancers and benign lesions were insignificant

Ten studies regarding D* value used in differentiating lung tumors were included for analysis. The χ^2^ = 55.48 and P < 0.001 of heterogeneity test with I^2^ = 84% suggested obvious heterogeneity among included studies. The forest plot in Fig. 6 showed the distribution of D* between lung cancer and benign lesions. A random-effects model generated a SMD of 0.01 (− 0.40, 0.42) (*P* = 0.96) between lung cancer and benign lesions for D*. A basically symmetric funnel plot in Fig. 4 and *P* = 1.00 of Begg’s Test suggested no publication bias in D*.

#### f value used for diagnosis of lung tumor

**Fig. 7:**
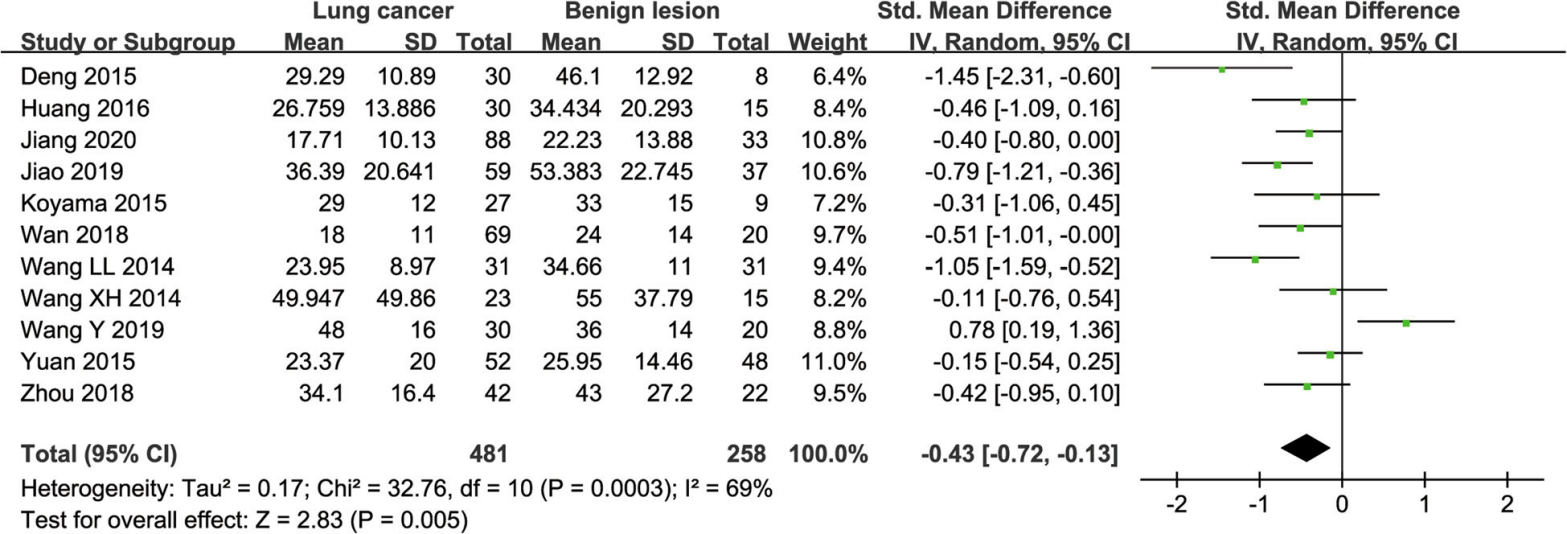
Forest plot of the mean value of perfusion fraction (f) between lung cancer and benign lesions. The standardized mean differences indicated that lung cancer had a significantly lower f value than benign lesions

Eleven studies regarding f value used in differentiating lung tumors were included for analysis. The χ^2^ = 32.76 and *P* < 0.001 of heterogeneity test with I^2^ = 69% suggested moderate heterogeneity among included studies. The forest plot in Fig. 7 showed the distribution of f value between lung cancer and benign lesions. A random-effects model generated a SMD of − 0.43 (− 0.72, − 0.13) (*P* = 0.005) between lung cancer and benign lesions for f value. A basically symmetric funnel plot in Fig. 4 and *P* = 0.640 of Begg’s Test suggested no publication bias in f value.

### Diagnostic performance

**Fig. 8:**
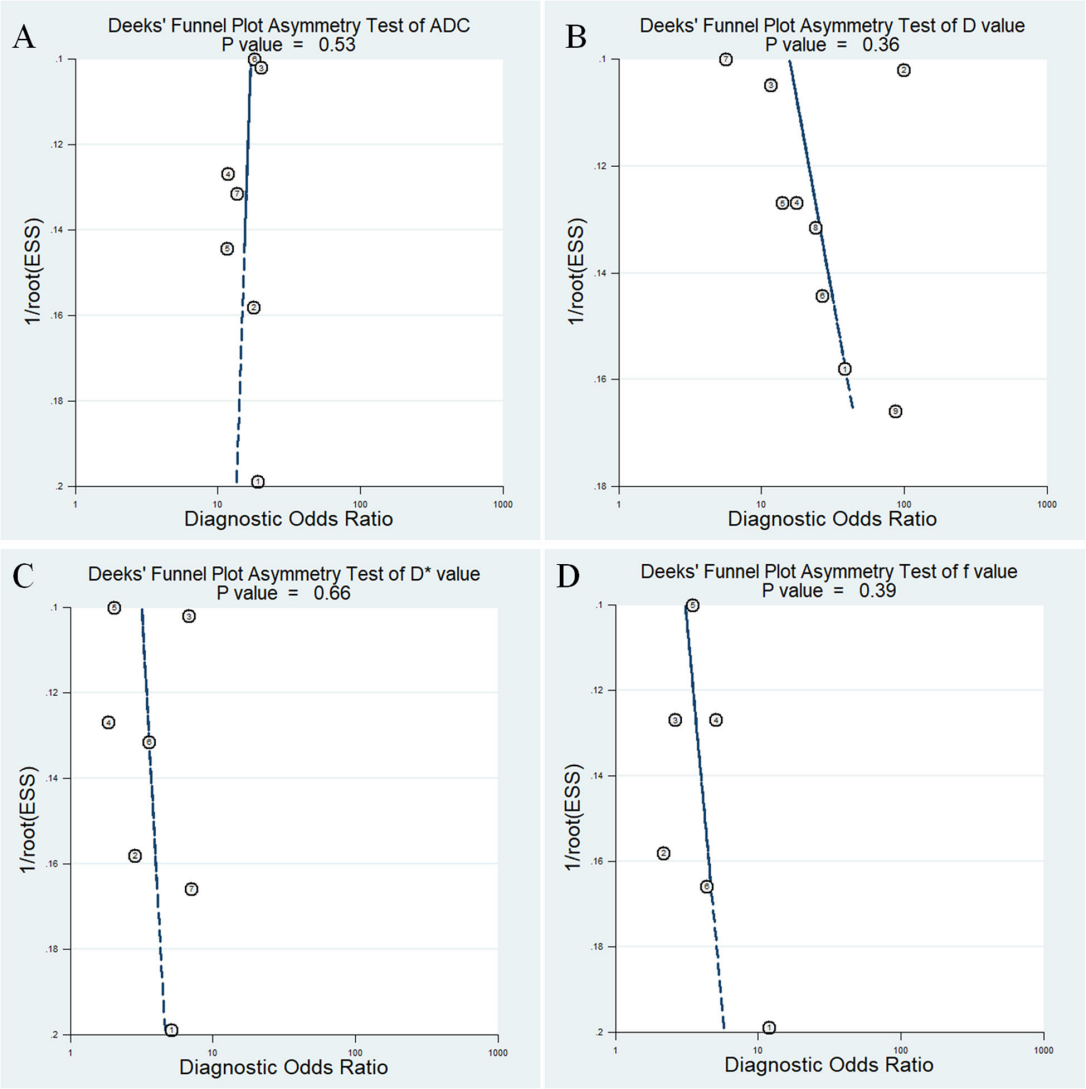
Deeks’ funnel plots regarding diagnostic performance for **a** apparent diffusion coefficient (ADC), **b** tissue diffusivity (D), **c** pseudo-diffusivity (D*), and **d** perfusion fraction (f). No publication bias was indicated in the four parameters (*P* > 0.05)

**Fig. 9:**
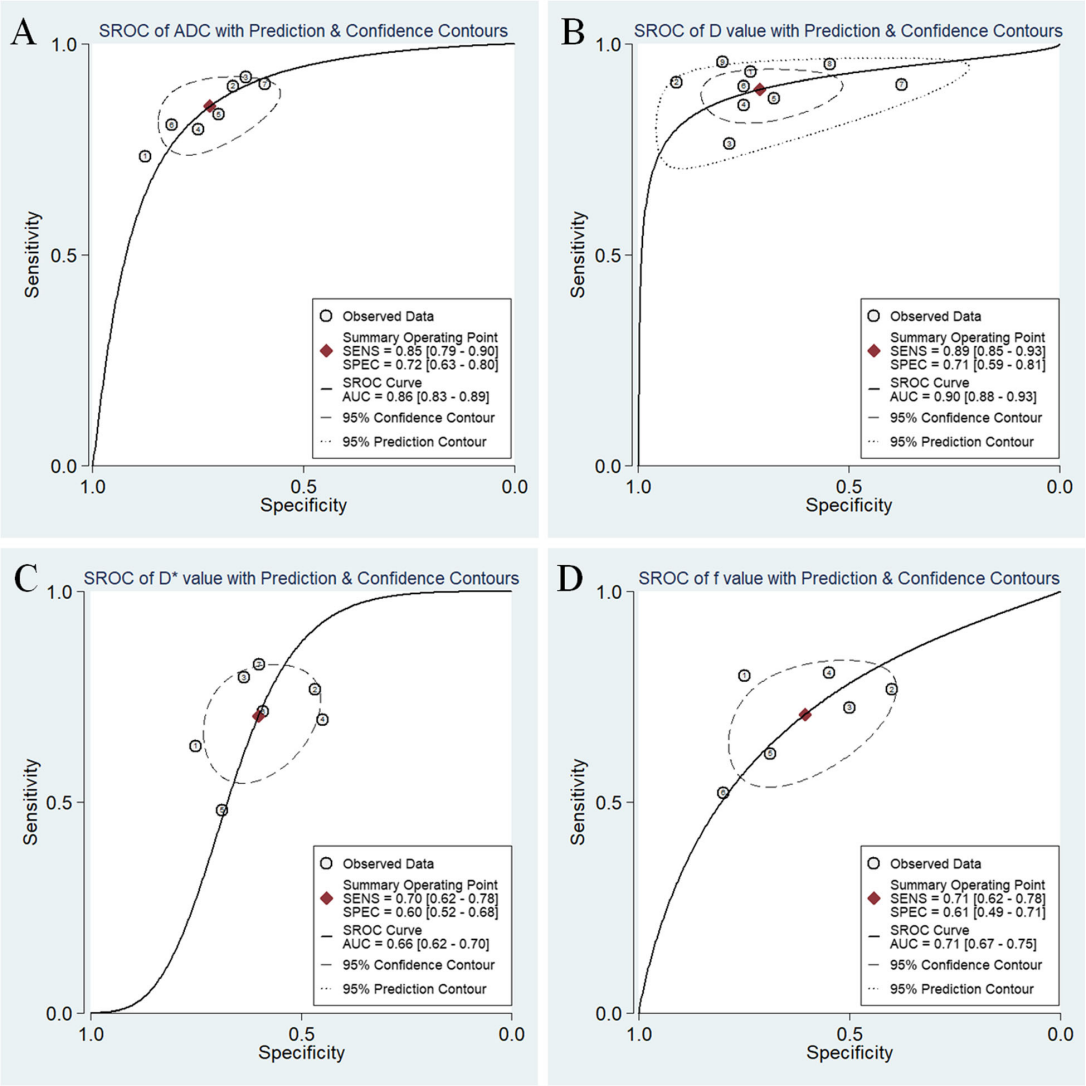
Summary receiver operating characteristic (SROC) curve of **a** apparent diffusion coefficient (ADC), **b** tissue diffusivity (D), **c** pseudo-diffusivity (D*), and **d** perfusion fraction (f) in the diagnosis of lung lesions. D value demonstrated the highest area under the curve, followed by ADC, f and D* values

The Diagnostic performance with pooled sensitivity, specificity, PLR, NLR, DOR and AUC of ADC, D, D* and f values were listed in Table 3. Deek’s funnel plots in Fig. 8 and asymmetry tests indicated no obvious publication bias in ADC, D, D* and f values (*P* = 0.53, 0.36, 0.66 and 0.39 for ADC, D, D* and f values, respectively). Fig. 9 plotted the summary receiver operating characteristic curves of ADC, D, D* and f values. D value demonstrated the best diagnostic performance (sensitivity = 89%, specificity = 71%, AUC = 0.90) in the differential diagnosis of lung tumors**,** followed by ADC (sensitivity = 85%, specificity = 72%, AUC = 0.86), f (sensitivity = 71%, specificity = 61%, AUC = 0.71) and D* values (sensitivity = 70%, specificity = 60%, AUC = 0.66).

**Table 3:** Pooled estimates and heterogeneity measures for ADC, D, D* and f values

Index	Sensitivity	Specificity	PLR	NLR	DOR	AUC	I^2^ (%)	
							Sensitivity	Specificity
ADC	0.85 (0.79,0.90)	0.72 (0.63,0.80)	3.1 (2.3,4.1)	0.20 (0.15,0.28)	15 (9,24)	0.86 (0.83,0.89)	43.07	3.91
D	0.89 (0.85,0.93)	0.71 (0.59,0.81)	3.1 (2.1,4.5)	0.15 (0.10,0.22)	20 (11,38)	0.90 (0.88,0.93)	44.52	77.62
D*	0.70 (0.62,0.78)	0.60 (0.52,0.68)	1.8 (1.4,2.2)	0.49 (0.37,0.65)	4 (2,6)	0.66 (0.62,0.70)	68.04	0
f	0.71 (0.62,0.78)	0.61 (0.49,0.71)	1.8 (1.4,2.3)	0.48 (0.37,0.62)	4 (2,6)	0.71 (0.67,0.75)	45.99	40.89

*ADC* Apparent diffusion coefficient, *D* Tissue diffusivity, D* Pseudo-diffusivity, *f* Perfusion fraction, *PLR* Positive likelihood ratio, *NLR* Negative likelihood ratio, *DOR* Diagnostic odds ratio, *AUC* Area under the curve; I^**2**^, inconsistency index

### Post-test probabilities

**Fig. 10:**
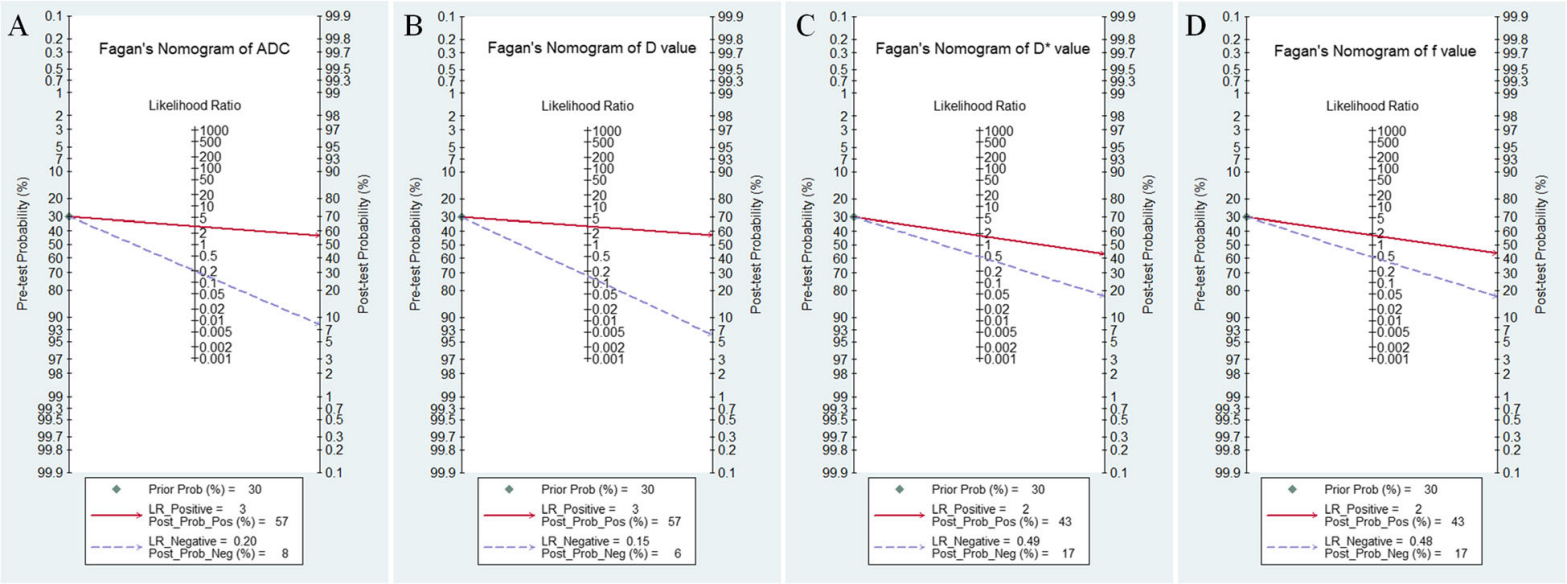
Fagan’s nomogram of **a** apparent diffusion coefficient (ADC), **b** tissue diffusivity (D), **c** pseudo-diffusivity (D*), and **d** perfusion fraction (f). D and ADC demonstrated similar and highest post-test probability among the four parameters

Likelihood ratio and post-test probability were also important for diagnosing a disease [21], which provided a likelihood that a patient was diagnosed with a certain disease or not using the MRI parameters. Fig. 10 plotted the Fagan’s nomograms of ADC, D, D* and f values for predicting post-test probabilities. All the pre-test probabilities were set at 30% by default. We regarded the diagnosis of lung cancer as a positive event, corresponding to a lower ADC, D and f values. Similarly, the noncancerous tissues with a higher ADC, D and f values were regarded as a negative event. The post-test probability increased to 57% from a pre-test probability of 30% with a PLR of 3.1 and decreased to 8% with a NLR of 0.20, with the prompt of ADC. This indicated that the diagnostic preference to lung cancer will be obviously enhanced with the help of ADC (a lower ADC) compared with the condition without the prompt of ADC whose diagnostic probability was set at 30% beforehand. In contrast, the probability of diagnosing lung cancer will significantly drop from 30 to 8% when a negative event occurs (a higher ADC). Similarly, the post-test probability of diagnosing lung cancer will reach to 57% with a PLR of 3.1 and drop to 6% with a NLR of 0.15 using D for guiding. The post-test probability of diagnosing lung cancer will reach to 43% with a PLR of 1.8 and drop to 17% with a NLR of 0.48 in the help of f value. These data indicated that both ADC and IVIM parameters helped to enhance the accuracy for diagnosing lung cancer.

## Discussion

IVIM-DWI is a noninvasive technique that shows superiority in reflecting tumor cellularity and perfusion without the need of contrast agent. It had already been applied in the differentiation of thyroid nodules [22], breast [23], liver [24] and brain tumors [25] with good diagnostic performance. To our best knowledge, there is still no pulmonary study with large sample size to settle down the value of IVIM for quantitatively distinguishing lung cancer from benign tissues, in the background of IVIM becoming a research hotspot in the whole-body tumors. Our study provided a timely summary in this issue through pooling all published evidence with strict inclusion criteria and quality assessment. The results demonstrated IVIM model had a good diagnostic performance in distinguishing lung lesions.

In this meta-analysis, the SMDs suggested that lung cancer demonstrated a lower ADC and D values than benign lesions. The lung cancer usually has dense cellularity and nucleoplasm ratio with active proliferative capacity, which may reduce the extracellular space and restrict the movement of water molecules, causing a reduction in diffusion coefficient. The pooled results also suggested an excellent diagnostic performance with a high sensitivity, specificity, AUC and increased post-test probability in both ADC and D values, followed by f value. Monoexponential model cannot provide an independent perfusion-related parameter and may miscalculate the water molecule movement due to a mix with microcirculation perfusion, and therefore resulted in an overestimated ADC value in a certain extent [23]. Therefore, the best diagnostic performance was observed in D value instead of ADC value.

Interestingly, lung cancer demonstrated a significant lower f value but insignificant D* value compared with benign lesions. F value refers to vascular volume ratio and reflects the microcirculation perfusion in the capillaries. F value increases with increased tissue perfusion. Higher f value is supposed to be observed in malignant tumors due to neovascularization, compared to benign lesions. However, these results are not unreasonable because the benign lesions occurring in the lung are generally inflammatory infections which consist of tuberculosis, organic pneumonia, fungal infection, granuloma or blood-rich tumor such as inflammatory pseudotumor. They are usually featured by marked vascular changes, including vasodilation, increased blood flow and enhanced vessel permeability, which generally occur at the capillary network [7]. A perfusion study using CT with exogenous contrast indicated active infectious nodules had comparable or even higher perfusion, peak enhancement increment, and blood volume with steeper time to peak than malignant nodules [26]. The results were in good agreement with our study in another aspect. However, the diagnostic performance of f value was relatively low with the sensitivity, specificity and AUC of 0.71, 0.67 and 0.71 only. F value is also associated with echo time, relaxation effects and T2 contribution [27], which may reduce its diagnostic accuracy/performance to a certain extent.

D* value is proportional to the average blood velocity and mean capillary segment length [28]. D * value was not statistically significant in differentiating benign and malignant lung lesions in this meta-analysis. A poor measurement reproducibility of D* was indicated by the huge standard deviations in the included studies. Theoretically, the more b-values are selected, the higher the accuracy of model fitting will be. Besides, measurement at lower b-value had been reported to be less reproducible and stable compared with measurement at higher b-value, and previous studies suggested measurements at a larger number of lower b-value should be obtained for reducing measurement errors and signal-to noise variation [29, 30]. However, a larger number of b-value applied in IVIM model will significantly prolong the scanning times and introduce obvious motion and susceptibility artifacts, especially in the pulmonary MRI. Therefore, D* value is still not adequate to differentiate lung lesions due to the low reliability, stability and accuracy, as indicated in our meta-analysis.

ADC, D, D* and f values all demonstrated moderate to obvious heterogeneity, which should be explored. First, both 1.5 T and 3.0 T MR scanners with various combinations of b-value were used to perform IVIM-DWI in these studies, which may influence the accurate calculations of diffusion and perfusion coefficients, and decrease the diagnostic performance compared to mono-exponential ADC. Second, the lesion sizes and density of lung cancer (such as ground glass opacity) on initial CT varied from studies to studies, which may perform different biological characteristics and also lead to the measurement variability in ADC and IVIM parameters indicated by Weller et al. [31] and Jiang et al. [32]. Third, the benign lesions consisted of a variety of inflammatory infections and benign tumors, which may introduce significant heterogeneity in these parameters when compared with lung cancer. Last, most studies delineated the regions of interest on the largest slice instead of the entire tumors, which may lead to some selection bias owing to tumor heterogeneity. Histogram analyses for the whole lesions, which can reduce the measurement variability, may be a more promising method for assessing lung nodules in the future study.

There were several limitations. First, as the sensitivity of detecting pure ground glass opacity or small lesions are quite low on conventional DWI or IVIM-DWI, these lesions were inevitably excluded from the original studies, which may decrease the availability of IVIM in the clinical application to a certain extent. Second, we had not performed a direct comparison with dynamic contrast enhanced-CT/MRI or Fluorine 18-FDG PET-CT, which was also commonly used in the diagnosis of lung cancer. The issue about whether IVIM-DWI added values to multi-parametric MRI or CT in a large sample size was still not clear**.**

## Conclusions

IVIM-DWI parameters show potentially strong diagnostic capabilities in the differential diagnosis of lung tumors, and D value demonstrated better diagnostic performance compared to mono-exponential ADC. F value can differentiate the perfusion difference between lung cancer and benign lesions. The application of IVIM-DWI will further help the clinicians make a better management for cancer treatment and prognosis evaluation based on the tumor cellularity and perfusion characteristics detected by IVIM technique.

## Data Availability

All the original data were provided in the main document, as well as the tables and figures. They can also be obtained from the Internet databases.

## Abbreviations

AUC: Area under the curve
ADC: Apparent diffusion coefficient
D: Tissue diffusivity
D*: Pseudo-diffusivity
IVIM-DWI: Intravoxel incoherent motion diffusion-weighted imaging
SMD: Standardized mean difference
I^**2**^: Inconsistency index
PLR: Positive likelihood ratio
NLR: Negative likelihood ratio
DOR: Diagnostic odds ratio
